# A Unique Case Linking Rituximab to a Ureaplasma Infection and Life-Threatening Hyperammonemia

**DOI:** 10.7759/cureus.74426

**Published:** 2024-11-25

**Authors:** Muhammad Muneeb Ibad, Samreen Jawaid, Sowmya Manjari Siddenthi, Aliyan Warraich, Omar Rahman

**Affiliations:** 1 Pulmonary & Critical Care, Indiana University Health Methodist Hospital, Indianapolis, USA

**Keywords:** granulomatosis with polyangiiitis, hyperammonemia, hyperammonemia-encephalopathy, immunosuppressant, non-cirrhotic hyperammonemia, rituximab, shock, ureaplasma, ureaplasma urealyticum, wegener's granulomatosis

## Abstract

Rituximab is an anti-CD20 monoclonal antibody medication used in treating various cancers like non-Hodgkin lymphomas as well as immunologic conditions like granulomatosis with polyangiitis. It disrupts and decreases the number of B-cells, which causes an immunosuppressive state. This can promote the growth of numerous rare and opportunistic pathogens, one of which is *Ureaplasma*. Increasing cases of *Ureaplasma* infections have been reported with rituximab use, but they do not typically present with hyperammonemia.

This is a case of widespread *Ureaplasma urealyticum* infection with shock, polyarticular arthritis and life-threatening hyperammonemia (ammonia levels greater than 160 microlmol/L) after rituximab use for granulomatosis with polyangiitis. It draws attention to the crucial point that early recognition of symptoms and timely diagnosis can lead to improved patient outcomes. Ureaplasma and other opportunistic organisms must be considered when reviewing patients on immunomodulating therapy.

## Introduction

The genus *Ureaplasma* belongs to the class Mollicutes, order Mycoplasmatales and family *Mycoplasmataceae* [1]. The organisms were first called T(tiny)-strains as they produced tiny colonies. Then they were later called T-mycoplasmas or T-strain mycoplasmas. It was discovered in the mid-1960s that they are unique among the mycoplasmas of human origin in that they metabolise urea. This discovery formed the basis of the metabolism-inhibition test that is developed to detect antibodies to them [2].

*Ureaplasma urealyticum* and *Ureaplasma parvum* are small free-living organisms that colonise the genito-urinary tract [3,4]. The first documented isolation of *Ureaplasma* was in male patients experiencing non-gonococcal urethritis (NGU) [5]. Since then, additional evidence has accumulated implicating *Ureaplasma* in infertility, postpartum endometritis, chorioamnionitis, spontaneous abortion, stillbirth, premature birth, perinatal morbidity and mortality, pneumonia, bacteremia, meningitis, and bronchopulmonary dysplasia (BPD) [4].

The major virulence factor associated with *Ureaplasma* is predicted to be multiple-banded antigen (MBA) and it is the major antigen recognised by sera during infections [6]. It undergoes a high rate of variation in vitro and may be involved in stimulation of the host inflammatory response [7]. Comparative genome analysis suggests *Ureaplasma urealyticum* is more capable of acquiring genes horizontally, which may contribute to its greater virulence for some conditions [8]. *Ureaplasma parvum* is more common than *Ureaplasma urealyticum* as a colonizer of the male and female urogenital tracts and in the neonatal respiratory tract [9].

All Mollicutes lack a hard cell wall, which prevents them from Gram staining, gives their cells pleomorphism, and makes them extremely vulnerable to dehydration. This causes a short life span in vitro which makes them difficult to detect [4].

In immunocompromised conditions like haematological conditions, immunosuppressive therapies, and organ transplant recipients, these organisms can disseminate and cause extragenital infections [10]. Here we describe a case of hyperammonemia secondary to disseminated *Ureaplasma* *urealyticum* in a young woman on rituximab therapy for her long-standing granulomatosis with polyangiitis.

This case report was previously presented at the Society of Critical Care Medicine’s (SCCM) 2023 Critical Care Congress.

## Case presentation

In May, 2022 a 22-year-old female patient presented to the Emergency Department with chest pain, bilateral knee pain, left shoulder pain, nausea, vomiting and shortness of breath. She had a past history of granulomatosis with polyangiitis since 2012 and had been managing that with rituximab for the past year. She also suffered from chronic renal failure for which she underwent hemodialysis and had past history of multiple urinary tract infections (UTIs).

**Figure 1:**
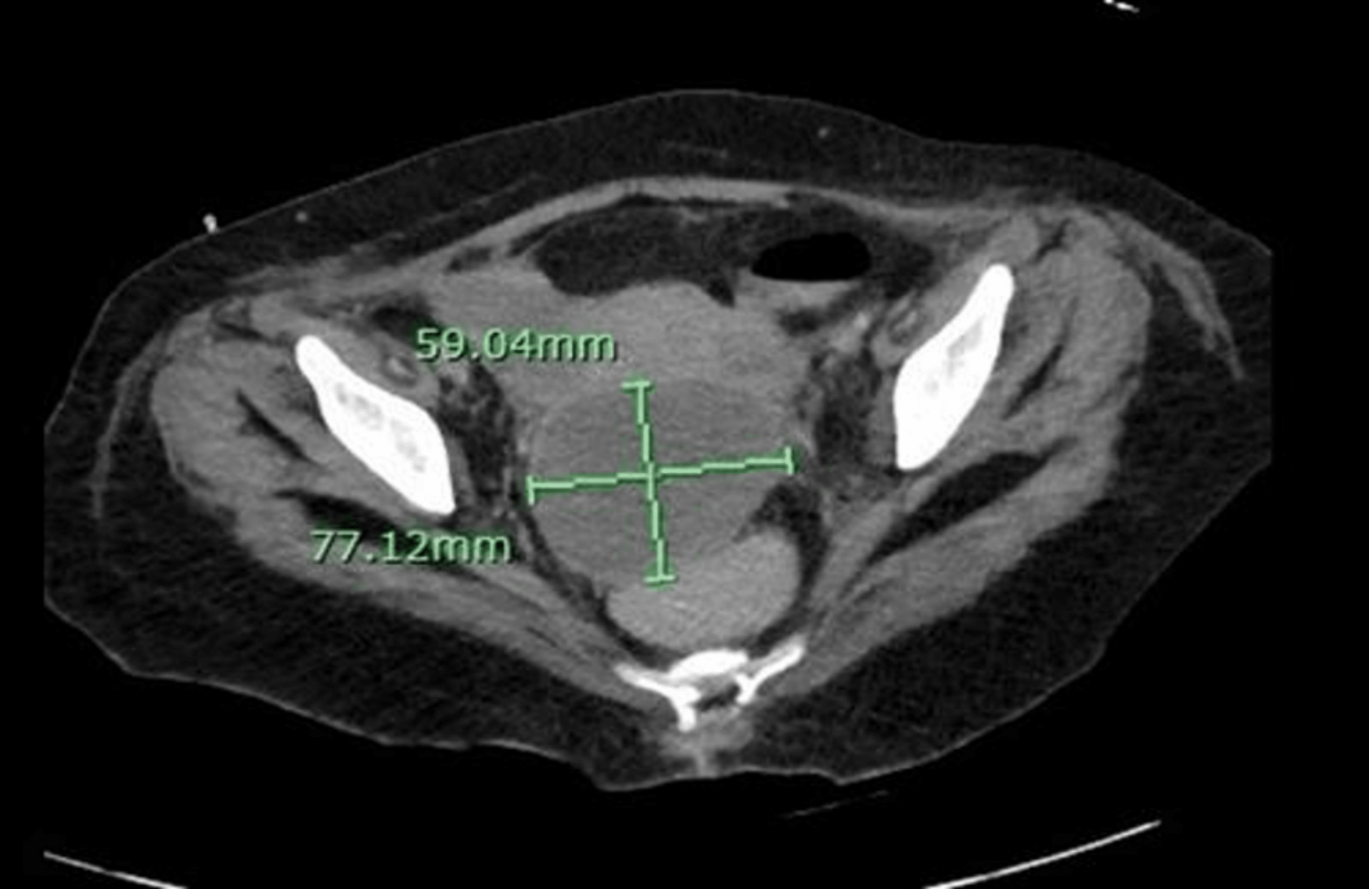
CT scan showing a paracoccygeal abscess

**Figure 2:**
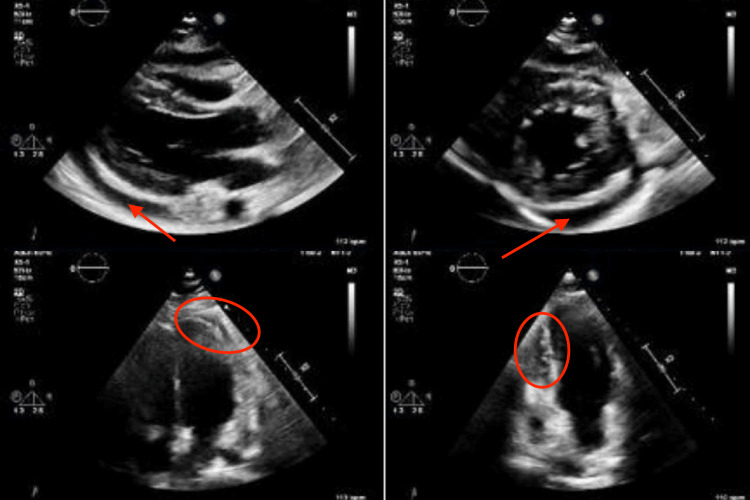
An echocardiogram showing areas of fluid collection in the pericardial cavity. The effusion is indicated by the red circles and the red arrows.

Her condition deteriorated quickly as she went into shock a day after admission. She had to be shifted to the ICU as she deteriorated and required intubation and vasopressors. A CT scan was performed which showed a small paracoccygeal abscess and polyarticular arthritis (Figure 1). Meanwhile, an echocardiogram was ordered showing a pericardial effusion (Figure 2).

Her liver function tests and lactate levels were elevated but then her signs and symptoms started improving with CT-guided pelvic abscess drainage and pericardiocentesis. She also underwent washout of the septic right hip joint and both the knee joints. She was taken off vasopressors and sedation after six days and was extubated five days later.

She was transferred back to the ward as her signs and symptoms improved. However, she then had to be transferred back to the ICU after being on the ward for two weeks due to slowly decompensating throughout the week as her ammonia levels rose back up and she developed reduced consciousness. She had been started on broad-spectrum antimicrobial treatment without noted improvement and her body fluid cultures were without growth.

She developed hyperammonemia with levels reaching as high as 361 mmol/L and was noted to be resistant to the various therapies given which included lactulose, rifaximin and L-carnitine. This raised concern for a urea cycle disorder and appropriate tests for it were ordered. She also had a bedside liver biopsy done on the same day which showed diffuse hepatocellular clearing/pale cytoplasm raising the possibility of a glycogenic hepatopathy. Her tests returned negative for urea cycle disorder testing.

**Figure 3:**
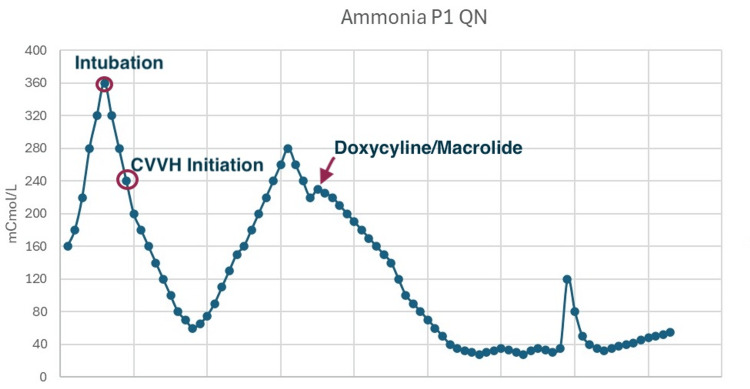
Graphical representation of the ammonia levels during the stay in the hospital. The effectiveness of continuous veno-venous hemofiltration (CVVH) can be contrasted and compared to that of doxycycline and azithromycin.

Her condition kept worsening with increasing deterioration of her mental status and she was subsequently started on continuous veno-venous hemofiltration (CVVH) without significant improvement in her ammonia levels, therefore requiring intubation for her declining mental state. The next day she was started on CVVH. Despite the ammonia levels initially decreasing, they never went back to normal (Figure 3).

A month after initial presentation she began receiving empiric treatment for a possible *Ureaplasma* infection with doxycycline (100mg oral suspension once daily) and azithromycin (500mg one tablet daily). This led to an overall improvement and eventual resolution of the encephalopathy and hyperammonemia. Meanwhile the polymerase chain reaction (PCR) for *Ureaplasma urealyticum* came back positive in both the respiratory and urinary samples. 

She gradually kept on improving and was finally transferred out to the ward almost one and a half months after initial presentation. She continued to receive azithromycin and doxycycline in the ward for an additional two weeks.

## Discussion

We discuss one of the rare instances when an invasive *Ureaplasma* presented with a wide range of symptoms. The patient initially presented with shock and later presented with refractory hyperammonemic encephalopathy, pelvic (paracoccygeal) abscess, pericardial effusion and polyarticular septic arthritis. To the best of our knowledge such a widespread infection with severe life-threatening hyperammonemia, polyarthritis and shock has not been reported before for a *Ureaplasma urealyticum* infection in a patient who was on rituximab.

While *Ureaplasma* has a propensity to cause abscesses [11], septic arthritis [12] or genitourinary diseases [13], invasive infection is much rarer and is seen mostly in immunocompromised or immunosuppressed patients [14]. It can be noted that all the cases referenced had the patients receiving rituximab as part of their therapy and in three of these cases [12-14] rituximab was taken in the past 12 months.

Previously *Ureaplasma urealyticum* infection was mostly associated with congenital immune diseases but the increased use of immunosuppressants including rituximab in recent times has led to an increase in the incidence of *Ureaplasma* infections [12]. It is pertinent to note that off-label use of rituximab for autoimmune kidney diseases and renal transplant has also increased in recent years [15,16].

Rituximab is an anti-CD20 monoclonal antibody that decreases the number of B cells. This has been associated with an increase in infections [16], including life-threatening infections. Pulmonary infections were the most common type observed according to this study looking into severe infections following rituximab use [17] while a past study observed hepatitis B infection as the most common viral infection in patients being treated for lymphoma with rituximab [18].

*Ureaplasma* is part of the normal flora, colonizing the urogenital tract lining in females. It contains the enzyme urease which causes the formation of vast amounts of urea which leads to hyperammonemia [19]. It is detected through PCR of body fluids and does not show up on gram stain or culture growth. All this makes diagnosing *Ureaplasma* infections challenging, delaying the start of the appropriate antibiotics. Similarly, as they lack a cell wall, they are resistant to beta-lactam antibiotics which are usually given as an empiric antimicrobial therapy. They have also shown resistance to sulfonamides based on laboratory testing [13]. All these factors of delayed diagnosis and appropriate treatment combine to cause a very high rate of mortality [19].

We agree with the conclusions given by Madlener et al. [20]. It is crucial that these rare infections are considered as differential diagnoses when dealing with patients on long-term immunosuppressants or humoral immunodeficiencies who fail to respond to initial antibiotic therapy and show no organisms on Gram stain or culture. 

## Conclusions

Prolonged immunosuppression can cause rare infections, including by those organisms that do not show up on routine diagnostic testing. *Ureaplasma* is one of these organisms and has an increased tendency to show up in transplant recipients and now increasing cases have been reported in patients with rituximab use. Thus, the possibility for such etiology must be kept in mind in immunosuppressed patients. *Ureaplasma* shows increased sensitivity to doxycycline and macrolides- specifically azithromycin and clarithromycin. Workup for *Ureaplasma* should be especially considered with negative Gram stain and cultures as well as when there is no response to standard antibiotic therapy. Ureaplasma should be kept in mind when dealing with high ammonia levels or in a patient on immunosuppressants.
